# Treating the unsayable: Pain and the problems of language

**DOI:** 10.1017/S1478951525101314

**Published:** 2025-12-29

**Authors:** Aldis H. Petriceks

**Affiliations:** 1 Department of Medicine, Beth Israel Deaconess Medical Center, Boston, MA, USA; 2 Harvard Medical School, Boston, MA, USA

## Manuscript text

Early in my residency, at that stage where I was meeting clinic patients for the first time, I entered the examination room of a man from Latin America. He had been a patient of my predecessor’s and suffered from complex chronic pain. I spoke fluent Spanish and introduced myself as his new primary care doctor. As though a prayer had been answered from the heavens, he threw his hands up and exclaimed ‘*¡gracias a Dios!*’

When I first met this patient, I could tell from his chart that many of our visits would center on pain management. I knew, also, that I could not perfectly manage his pain at the very beginning of our time together – and at the very beginning of my training in primary care and internal medicine. I referred him to our pain clinic, and did my best to understand and treat his pain in the intervening time.

This involved the usual questions about pain – its nature, its onset and course, prior interventions, triggers, goals. I had the fluency and medical vocabulary to discuss these things in Spanish, but as our visits progressed, I was bothered by a vague discomfort. I understood everything the man was telling me, but I could not connect with what was being said.

I don’t mean that I didn’t sympathize. Nor that I hadn’t had such experience of pain (though that would be true). Beyond syntax or sympathy, there was something in the language that did not *hit* me. There was some lack of visceral response, even if my emotions were triggered by what the man had told me about his pain.

There was an easy explanation: I am a fluent, but not a native, speaker of Spanish. The language does not live in my bones the way it would had I grown up in, say, Medellín, Colombia, where I completed a psychiatry rotation during medical school. (There, a patient once told me in somewhat poetic fashion: *no tengo ninguna enfermedad mental. Es la vida cotidiana que me golpeó, o contra la que me he golpeado.* ‘I don’t have a mental illness. It is everyday life that struck me, or against which I have struck myself.’ I understood his meaning perfectly.) But that explanation is almost too easy, and obscures a troubling question: would I have understood this man’s pain had he reported it to me in English?

In her influential book, *The Body in Pain: The Making and Unmaking of the World* (1985), the philosopher Elaine Scarry casts doubt on that question. Scarry marks pain as the most immediate of human experiences, but only for the sufferer. ‘For the person whose pain it is’, she argues, ‘it is ‘effortlessly’ grasped (that is, even with the most heroic effort it cannot not be grasped)…’ (Scarry 1985). But the onlooker or would-be healer is unable to share in this experience, no matter how excruciatingly described. ‘Whatever pain achieves’, Scarry continues, ‘it achieves in part through its unsharability, and it ensures this unsharability through its resistance to language’.

For Scarry, the disconnect between what my patient was saying and what I was feeling was not necessarily a failure of empathy or imagination. Some version of that disconnect would remain had the patient and I grown up in the same apartment complex in Latin America or Northern California. Scarry quotes Virginia Woolf, who claims that ‘English… which can express the thoughts of Hamlet and the tragedy of Lear has no words for the shiver or the headache’, and so Shakespeare may suffice across centuries for the most sublime human emotions but ‘let a sufferer try to describe a pain in his head to a doctor and language at once runs dry’(Scarry 1985).

Of course, even a resident in internal medicine, far from being an expert in pain management, can tell you that language rarely runs dry. Language persists, but it comes out collaterally – sometimes exasperated, sometimes dysphoric, often pragmatic. This is as true in English as it is in Spanish, or in Mandarin, which I also speak with patients. In each of these languages, far more urgent than a desire to communicate the incommunicable is a patient’s need to find help. (I recall speaking Mandarin to a patient with urinary retention in the emergency department, who urged me to stop with all the useless talk and place a urinary catheter to relieve their obstruction.)

But in my experience, particularly in complex chronic pain, finding help can be a temporary intervention, a way to buy time. The next visit, the next hospitalization, still throws us back on one of the many problems of pain: for all its universality, does pain separate more than it connects? The question is not so far from another troubling doubt: does *language* separate more than it connects?

Pain is a physical experience and can only be expressed verbally in metaphor or simile. We may take cultural and linguistic differences to be the source of misunderstanding in our assessment of pain across cultures, but this realization does not offer a ready-made resolution. Rather, as Scarry argues, ‘such cultural differences… expose and confirm the universal sameness of the central problem’ of understanding and communicating pain: ‘a problem that originates much less in the inflexibility of any one language or in the shyness of any one culture that in the utter rigidity of pain itself’, so that pain’s incommunicability ‘is essential to what it is’ (Scarry 1985). Not only is language insufficient to truly communicate pain, it is in some ways an obstruction to such communication.

Words are the medium in which we seek to understand pain, but they also alienate, precisely because pain does not reside or originate in words. That alienation grows when I ask whether I *really* want to understand another’s pain. This would involve a physical replication of their suffering in my own body, which I obviously do not want. And even then, would I finally understand what they are going through? What their pain means *to them*?

To pull this back from the threat of solipsism, I’m brought to language itself. Language is a tool for communication and mutual understanding, as well as a dividing line. I feel that you are different from me because you speak another language; evolution and change are central to the genetics of speech; and words are used to describe as well as to deceive or deride. Yet none of this invalidates speech, nor the effort to connect with others through speech. There is going to be some misunderstanding when individuals use words together, even when they are speaking the same language. But what comes across, what we want to come across, is an adequate dose of truth and (what is equally important) an acknowledgement and care for the person with whom I speak.

There is going to be a fundamental distance between myself and a patient suffering from pain. This distance is on a different axis from other kinds of separation. It exists even if the patient and I have a strong clinical rapport. It would exist if the roles were reversed, or if we were both in pain. But my understanding of the patient’s pain is still enriched by attention to how they describe that pain. Not only because this helps me characterize pain as neuropathic, visceral, etcetera, but also because it helps me to care for them as an individual, feeling and expressing pain in their unique way.

Stepping back, this also helps me to see the fraught and shifting nature of my work. As a doctor, I’m trained to form clear plans, to diagnose and manage discreet conditions. But when it comes to pain and the meaning of pain, I have to see that I’m never quite grasping the whole of what’s happening. There’s always more to understand, even though I can’t force that understanding, just as I can’t force meaningful communication from anyone.

What I can do is create the conditions for meaning to arise, open spaces for the light to get in. This has as much to do with silence as it does with speech. Silence, not language, is what I turn to when pain bankrupts language. It is a kind of meaning in chorus with my patients, its own kind of prayer.

## References

[ref1] Scarry E (1985) *The Body in Pain*. New York, USA: Oxford University Press.

